# Innovation in Wood Preservation

**DOI:** 10.3390/polym12071511

**Published:** 2020-07-07

**Authors:** Roger M. Rowell

**Affiliations:** Biological Systems Engineering, University of Wisconsin, Madison, WI 53705, USA; rmrowell@wisc.edu

**Keywords:** moisture, preservative, mechanism, acetylation, decay, brown-rot fungus, wood, equilibrium moisture content, toxicity, acetyl content, sugar analysis, weight loss

## Abstract

The wood preservation industry has depended on toxicity as a mechanism of effectiveness against decay fungi to extend the life of wood used in adverse conditions. An alternative to toxicity, however, is to study and understand the mechanism of fungal attack and stop it before it can start. Knowing that fungi need moisture for colonization, a new approach to wood preservation is to lower the cell wall moisture content below that needed for fungal attack. Acetylation chemistry is known to reduce the moisture content in the cell wall, and it was used to study moisture levels in the bulk cell wall and in the isolated cell wall polymers. Resistance to brown-rot was determined using a 12-week soil block test with *Gloeophyllum trabeum*. Weight loss was measured and an analysis of what was lost was determined.

## 1. Introduction

Wood has been used by humans since the first humans walked the earth. They used it for fuel, shelter, weapons, tools and for decoration. Since we have used and studied it so long, you would think that we knew all there was to know about it—but that is not the case. There is a lot of misunderstanding about wood. For example, we say wood is renewable and sustainable. But this is not true. A tree is renewable and in order to ensure a continuous supply of wood, management of agricultural and forest lands should be under a proactive system of land management whose goal is both sustainable forestry and the promotion of healthy ecosystems. We also say that wood was designed by Nature as a building material. This is also not true. Wood was designed to perform when wet so it is flexible to bend in the wind. We cut the wet tree down, dry it and use it for products that are durable. Also, not true. Wood was designed to degrade back to CO_2_ and water through five basic chemistries: Oxidation, Hydrolysis, Reduction, Free Radical, and Dehydration. Can you imagine what the world would look like if wood did not decay? Finally, we refer to wood as a material. A scientific definition of materials is that it is consistent, uniform, predictable, and reproduceable. No two pieces of wood are alike even if they come from the same tree, the same board or within a board.

If a person wants to build, for example, a doghouse they will use wood. They know it will not last very long but neither will the dog! So, the perception of wood is that it is a common resource used by common people with limited expectations. How can we change that? How can we show the public that wood can be durable and last longer than the dog?

When we think of making wood last longer in the environment, we think of wood treated with a wood preservative. The wood preservation industry had depended on toxicity as a mechanism of effectiveness against decay for many years. For example, various formulations and combination of chromium copper and arsenic, borates, pentachlorophenol in various formulations and creosote. Many of these have either been banned in many countries or only allowed limited use.

However, there is another approach to wood preservation not based on toxicity.

Wood is degraded by fungi because these organisms have the ability to recognize the structure and break down the wood chemistry into digestible units. While the brown-rot fungi only accounts for about 6% of the total fungal population, it is the one that causes the most damage especially in softwoods. There are many theories on the mechanism of the attack of brown-rot fungi on softwoods. Many researchers have tried to explain the mechanism as being one or two steps. However, I think that is too simple and the actual mechanism involves many steps.

Theoretically, the first step in fungal attack may be a favorable environment. Fungi need an environment that is conducive to their survival: temperature, moisture, pH, and toxicity. If the temperature is too high or too low, the fungi cannot colonize. If the wood is too wet or too dry, the fungi cannot colonize. If the pH is too high or too low, the fungi cannot colonize. And, if there are toxic chemicals in the wood, the fungi cannot survive. If the environment is favorable, the fungal attack starts. It may start with some type of substrate recognition, but we see the pH of the wood starts to drop as the fungus colonizes. The hyphae of a brown-rot fungus must detect a source of nutrition in the wood it has come into contact with in order to survive. Early in the attack is the oxidation and rearrangement of lignin. No lignin is lost at this point, but it is known to be somewhat oxidized and condensed [1]. At this stage, the fungus is undergoing gene expression to start the production of cellulosic enzymes [2,3,4].

Since the result of early brown-rot degradation is a loss of cell wall carbohydrate polymers (mainly the hemicelluloses), it is logical to assume the first degrading reaction is a low molecular weight non-enzymatic system. One of the hemicellulose sugars that is lost in proportion to the loss of strength is arabinose [5]. Arabinose is the only hemicellulose sugar that exists in a strained five- membered pentose ring. It is possible that arabinose is involved in the early recognition of brown-rot attack on wood.

The first chemical reaction is the generation of a peroxide/ferrous ion and a hydroxyl radical chemical system that depolymerizes the structural polysaccharides in the cell wall matrix resulting in strength losses [6,7]. The hemicelluloses and the cellulose polymers start to

Fe⁺⁺ + H_2_O_2_ + H⁺ → Fe⁺⁺⁺ + ·OH (hydroxyl radical) + H_2_O

degrade, and major strength losses are observed due to the loss of the cell wall polymer matrix. As this Fenton chemistry progresses, a new enzyme reaction takes place in other accessible regions of the cell wall polysaccharides resulting in significant weight losses. Very little lignin is lost in the final stages of attack where the majority of wood weight is lost.

Moisture is part of the porous surface, it is needed for the production of oxalate and its movement in Fenton chemistry is necessary at a glyosidic bond for hydrolysis. It is needed for enzyme activity, generated by the fungus as a by-product in digestion, and, finally, it is needed to move soluble sugars. All of these are steps involve “wet” chemistry and the question is how much moisture is needed, where does it come from and where is it located in the cell wall? The key is to reduce the moisture content of the cell wall below that required for fungal attack.

Griffin reported that decay fungi are most active when the wood moisture content is between 40% and 85% moisture content [8]. In 1960, Stamm and Baechler reported that a moisture content above 20% is necessary for fungal attack [9].

## 2. Background

There are many publications on the effect of moisture on wood decay but they all deal with bulk properties of moisture in the cell wall not on specific moisture in the cell wall polymers [2,10,11,12,13,14,15,16,17,18]. The specific questions are: where in the cell wall is the moisture, how much is needed for decay and how does it move within the cell wall?

This paper is not intended as a review of all the data on moisture and decay in wood. That has been done several times and it would just add pages to this manuscript to repeat it. I have included all references that are relevant to understand the concepts presented.

There is also data on the weight loss in a fungal decay tests but no analysis of what was lost. It is well known that the hemicellulose polymers are the most hygroscopic component of the cell wall, so it is logical to assume that moisture in the hemicelluloses is the most accessible to the fungus [19].

One chemistry known to reduce cell wall moisture content is the reaction of wood with acetic anhydride or acetylation. I have done research on wood acetylation since 1975 and have published many papers on the subject [20]. Recently, however, I have started to study the correlation between wood decay test results and bulk cell wall moisture content, moisture in the cell wall polymers and moisture movement. One way to study reduction in cell wall moisture content is using the simple chemical modification system of acetylation.

Acetylation takes place in the most accessible hydroxyl groups in the cell wall polymers. It is a single-addition reaction, which means that one acetyl group is on one hydroxyl group with no polymerization. Thus, all the weight gain in acetyl can be directly converted into units of

WOOD–OH + CH_3_–C(=O)–O–C(C=O)–CH_3_ → WOOD–O–C(=O)–CH_3_ + CH_3_–C(=O)–OH Acetic anhydride → Acetylated wood → Acetic acid

hydroxyl groups blocked.

In a 1945 office report of the USDA, Forest Service, Forest Products Laboratory, Tarkow was the first to report that acetylated balsa was resistant to decay in a three-month soil test [21]. He did not describe the acetylation process or the acetyl content in that report but showed that acetylated balsa was resistant to attack by the brown-rot fungi *Poria incrassata* and *Poria microspore* and the white rot fungus *Polyporus versicolor*. After the three-month test, unreacted balsa lost an average of 7.8% weight loss with *Poria microspore* and 0.5% weight loss in the acetylated sample; 1% weight loss in unreacted balsa with *Poria incrassate* and 0.9% weight loss in the acetylated samples; 50.1% weight loss in unreacted balsa with *Polyporus versicolor* and 0.2% weight loss in the acetylated samples.

In a 1946 office report, Tarkow, Stamm, and Erickson described the acetylation process for balsa [22]. The reaction was done at 90 °C for 6 h in a mixture of acetic anhydride and 20% pyridine. The report that after three months in a soil test using *Poria incrassata* or *Poria microspore*, the control balsa lost 50% weight while the acetylated balsa showed no attack. The main purpose of this research was to study and determine dimensional of acetylated wood.

The purpose of the present research is to (1) report the correlation between bulk cell wall moisture content (EMC), moisture content of the individual cell wall polymers (SMC) and resistance to decay by brown-rot fungi on acetylated wood, (2) to report not only the weight loss due to brown- rot attack, but analyze what is lost, (3) view micrographs of the cell wall before and after fungal degradation to view the hypha growing in the cell wall.

## 3. Materials and Methods

Radiata pine or southern yellow pine samples 2.5 × 2.5 × 0.6 cm^3^, (radial × tangential × longitudinal) were reacted with acetic anhydride at 120 °C for various lengths of time to give different levels of bonded acetyl. Acetyl content was determined using wood oven-dry weight before and after the soil block test using gas chromatography according to the procedure outlined in Beckers et al. [23].

Equilibrium moisture content (EMC) was determined on oven-dry specimens with various acetyl contents placed in a constant humidity room at 65% or 90% relative humidity (RH) and 27 °C. After 21 days the specimens were weighed to determine the EMC. The EMC of separate specimens (2.5 cm^3^), that were used for the soil block test, were measured at 90% relative humidity (RH) and 27 °C before and after the soil block test. Three samples at each acetyl level were performed and the results averaged. The specific moisture content (SMC) of isolated lignin, cellulose, and hemicellulose was determined using the same method at 65% RH and 90% RH.

The ASTM D 1413 standard soil block test was performed on solid wood blocks (1.9 cm^3^) [24]. Untreated controls and acetylated wood were exposed to the brown-rot fungus *Gloeophyllum trabeum*. Specimens were removed from test after 12 weeks. The extent of decay was determined as oven dry weight loss.

Sugar content was determined both before and after the soil block test using HPLC according to the procedure outlined by Petterson et al. [25].

Lignin was isolated using the sodium chlorite/acetic acid method and the isolated holocellulose was separated into hemicellulose and cellulose fractions using dilute sodium hydroxide.

Scanning Electron Micrographs were taken of pine before and after brown-rot fungal attack.

## 4. Results

Table 1 shows the results of the acetylation reaction, the equilibrium moisture content before and after the soil block test and the weight loss in the 12-week brown-rot fungal soil block test. As expected, as the acetyl content increases, the EMC and the fungal attack decreases. There is a drop in the EMC at 90% RH after the soil block test. This may be to the loss of hemicellulose polymers which are more hydrophilic as compared to cellulose and lignin. It was not possible to determine the EMC of the control specimens at 90% RH after the soil block test because they were badly degraded and very little wood was left.

This data shows that the threshold to inhibit brown rot attack is approximately 20% acetyl which represents an equilibrium moisture content of the cell wall of approximately 8%. This is consistent with previously published data [26].

Table 2 shows the weight loss and carbohydrate analysis before and after the soil block test. At an acetyl level of 19% there is a total weight loss of 66% with only about 10% of the carbohydrate polymers left in the cell wall. There are major losses of all hemicellulose sugars at a weight loss of 5%. Some of the glucan loss may be due to hemicellulose glucose.

The lignin analysis did not change after the soil block test indicating that lignin was not degraded in the test.

Table 3 shows the EMC for whole wood and the specific moisture content (SMC) of each cell wall polymer measured at 90% RH. When we measure cell wall moisture it is an average moisture content of all cell wall polymers but does not give the specific moisture content of each individual polymer. The data in Table 3 show that the hemicelluloses have a much higher specific moisture content as compared to cellulose or lignin at both RH values.

Jakes et al. have reported that the glass transition temperature for hemicelluloses at 65% RH is 25 °C [27]. This means that at room temperature and 65% RH, the hemicelluloses are softened, and moisture is starting to percolate in the hemicellulose interconnecting matrix. The data in Table 3 shows that there is enough moisture in the hemicellulose polymers at 65% RH to support fungal colonization. 

**Figure 1:**
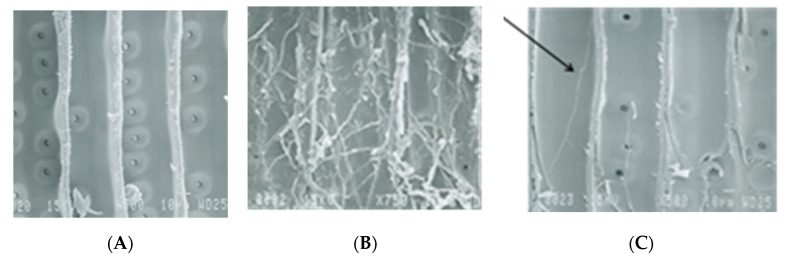
Electron micrographs of pine control before test (**A**), control after 12 weeks with a brown-rot fungus (**C**), and acetylated (**B**) after the 12-week test.

Figure 1 shows electron micrographs of control and acetylated pine. The control sample before the test (A) and the control after the 12-week test with a brown-rot fungus Figure 1B. The control sample after test is almost completely covered with fungal hypha with a destroyed cell wall. The acetylated sample (19 WPC) (1 C) shows a few hypha growing on the inner cell wall but very little weight loss is detected. Note in Figure 1C that the hypha is growing on the S_3_ layer of the inner cell wall which is high in lignin.

## 5. Discussion and Conclusions

There is a continuing need to extend the service life of wood used in adverse environments from decay and a need to use less toxic methods to protect the environment. Preservative treated wood is effective although not as effective as it was in the past and it must be disposed of in a toxic land fill.

One approach to a non-toxic method of wood preservation is to understand how a fungus recognizes wood as a food source and stop their attack before it can start. It is known that fungi need moisture. Their colonization chemistry requires moisture for the production of oxalate and its movement in the cell wall, in Fenton chemistry to degrade cell pall polysaccharides, for gene expression and enzyme activity, for glycoside hydrolysis, and for the movement of soluble nutrients.

This research has looked at the reduction of moisture in the cell was as result of acetylation. If the moisture content of acetylated wood in the cell wall is under 4%, the brown-rot fungus tested can not colonize. This level of reduced moisture content can be reached with an acetyl level of about 20%. But, the question is, where is the moisture located, how does it move, and how does the fungus access it. Looking at the specific moisture content of each cell wall polymer, it is found that the hemicellulose polymers have the highest moisture content (38%) as compared to lignin (16%) and cellulose (12%) at 90% relative humidity. It seems logical that the fungus accesses the moisture in the hemicelluloses which may be the first to be degraded. The hemicellulose polymers are inter-connected throughout the cell wall and can act as a hemicellulose moisture pipeline.

This study used on brown-rot fungus, *Gloeophyllum trabeum*. While a very active fungus for softwoods, it is only one of many that occur in Nature. A more realistic study is putting acetylated wood in out-door stake tests where there are many types of fungi, brown-, white-, and imperfect, are present. There are reports on in-ground tests on acetylated wood and at levels of 20% acetyl, the wood is protected for many years [28,29].

This research has also shown that acetylation is not toxic to the fungus as fungal hypha are visible growing on the intercell wall when no weight loss has been detected. This shows that the mechanism of effectiveness is not based on toxicity. It also shows that lignin acetylation may be more important than we have previously thought in that lignin is modified by the fungus in one of the early steps in the degradation process.

The exact mechanism of fungal resistance due to acetylation still needs more study but moisture is part of the answer. Not only the moisture content but where the moisture is in the cell wall as well as the accessibility of the hyphae tip to that moisture.

## Figures and Tables

**Table 1:** Acetyl content of radiata pine reacted with acetic anhydride at various reaction times at 120 °C, equilibrium moisture content (EMC) and weight loss in the 12-week soil block test.

Before Soil Block Test			After Soil Block Test				
Reaction Time	Acetyl	EMC at:	EMC at:	Weight loss	Acetyl	EMC at:	EMC at:
(hrs)	(%)	(%)	(%)	%	(%)	(%)	(%)
		65% RH	90% RH			65% RH	90% RH
0	0.63	9.4	21.7	65.8	0.22	-	-
0.25	5.8	7.0	16.8	58.8	4.8	7.5	11.8
0.5	11.0	5.2	14.4	44.9	10.5	6.9	10.3
1	14.8	4.6	11.4	35.6	13.7	6.2	9.7
2	19.8	3.7	8.4	5.0	18.9	4.6	7.2
4	24.8	2.1	3.2	>2	22.9	1.7	3.1

**Table 2:** Weight loss (%) and carbohydrate analysis before and after the 12 week soil block test.

Acetyl (%)	Total Weight Loss (%)	Total Carbon Lost (%)	Araban Lost (%)	Galactan Lost (%)	Rhamnan Lost (%)	Glucan Lost (%)	Xylan Lost (%)	Mannan Lost (%)
0	65.8	88.0	86.7	66.8	75.0	89.0	84.3	93.5
19	5.0	18.0	27.4	17.5	16.7	18.0	11.1	20.9

**Table 3:** Equilibrium moisture content and specific moisture content of whole wood and isolated cell wall polymers.

Sample	EMC at 65% RH	EMC at 90% RH
Whole wood	11	21
	SMC at 65% RH	SMC at 90% RH
Hemicellulose	22	38
Lignin	7	16
Cellulose	5	12
